# Late to the Party: Mental Health Professionals’ Knowledge on Party Drugs and Harm Reduction Advice

**DOI:** 10.1192/bjo.2022.126

**Published:** 2022-06-20

**Authors:** Nataly Gibson

**Affiliations:** South London and Maudsley NHS Foundation Trust, London, United Kingdom

## Abstract

**Aims:**

Knowledge of illegal substances has long revolved around addictions in psychiatry training and not of party drugs or harm reduction. Reasons for this could include it being a fairly taboo subject, and it being an area where information and advice change frequently. However, drug related deaths are at their highest since records began, and as our patients use them, it is important that professionals are knowledgeable and can offer sound harm reduction advice. The aims were to establish whether there was a deficit in mental health professionals’ knowledge and understanding of party drugs and harm reduction, to give education on this subject, and to gain feedback on whether it is useful and/or important.

**Methods:**

A questionnaire of 10 questions on party drugs and harm reduction was devised using resources from charities ‘The Loop’ and ‘Talking Drugs’. These questions aimed to test general knowledge in this area that would be expected from professionals. The study was carried out using Mental Health professionals (MDT) in a busy South London Trust in November 2019 and March 2020.The questionnaires were given before and after teaching sessions on the subject. Feedback was then collected from the attendees on their experiences.

**Results:**

Before the teaching sessions, professionals answered 44% of the questions correctly, 48% incorrectly, and 8% were ‘don't know’. However, after the sessions these scores went up to 77% correct, 19% incorrect, and 4% were ‘don't know’. Feedback was extremely positive, with an Addictions Consultant even commenting that she didn't know a lot of what was being taught! Professionals recognised the gap in their knowledge and were keen for more teaching.

**Conclusion:**

Party drugs and harm reduction knowledge is lacking in Mental Health professionals despite it being commonly seen in our patients. Informed, tailored teaching sessions can help improve this and it seems most professionals would welcome it. In the future it may be useful to include this type of teaching as part of the official Psychiatry curriculum.

